# Genetic diversity within and between British and Irish breeds: The maternal and paternal history of native ponies

**DOI:** 10.1002/ece3.5989

**Published:** 2020-01-27

**Authors:** Clare L. Winton, Robert McMahon, Matthew J. Hegarty, Neil R. McEwan, Mina C. G. Davies‐Morel, Charly Morgan, Deborah M. Nash

**Affiliations:** ^1^ Institute of Biological Environmental and Rural Sciences Aberystwyth University Aberystwyth UK; ^2^ Molecular Haematology Haematology Laboratory Royal Infirmary of Edinburgh Edinburgh UK; ^3^ School of Pharmacy and Life Sciences Robert Gordon University Aberdeen UK

**Keywords:** conservation, genetic diversity, mtDNA, ponies

## Abstract

The UK and Ireland have many native pony breeds with historical and cultural importance as well as being a source of uncharacterized genetic diversity. However, there is a lack of comprehensive research investigating their genetic diversity and phylogenetic interrelationships. Many studies contain a limited number of pony breeds or small sample sizes for these breeds. This may result in erroneous grouping of pony breeds that otherwise have intricate interrelationships with each other and are not evaluated correctly when placed as a token subset of a larger dataset. This is the first study that specifically investigates the genetic diversity within and between British and Irish native pony breeds using large sample numbers from locations of their native origin.

This study used a panel of microsatellite markers and sequence analysis of the mitochondrial control region to analyze the genetic diversity within and between 11 pony breeds from Britain and Ireland. A large dataset was collected (a total of 485 animals were used for mtDNA analysis and 450 for microsatellite analysis), and previously published data were used to place the British and Irish ponies in a global context.

The native ponies of Britain and Ireland were found to have had a complex history, and the interrelationships between the breeds were revealed. Overall, high levels of genetic diversity were maintained in native breeds, although some reduction was evident in small or isolated populations (Shetland, Carneddau, and Section C). Unusual mitochondrial diversity distribution patterns were apparent for the Carneddau and Dartmoor, although among breeds and global haplogroups there was a high degree of haplotype sharing evident, well‐represented within British and Irish ponies. Ancestral maternal diversity was maintained by most populations, particularly the Fells and Welsh ponies, which exhibited rare and ancient lineages. The maternal and paternal histories of the breeds are distinct, with male‐biased crossings between native breeds, and other shared influences, likely Arabs and Thoroughbreds, are apparent. The data generated herein provide valuable information to guide and implement the conservation of increasingly rare native genetic resources.

## Graphical abstract

This study is the first to specifically investigate the genetic diversity within and between British and Irish native pony breeds using large sample numbers from locations of their native origin and used a panel of microsatellite markers and sequence analysis of the mitochondrial control region to analyse the genetic diversity within and between 11 pony breeds from Britain and Ireland. The native ponies of Britain and Ireland were found to have had a complex history, and the interrelationships between the breeds were revealed. Overall, high levels of genetic diversity were present, including maintenance of rare ancient matrilines in the Welsh and Fell breeds. The study provides valuable information to guide and implement the conservation of increasingly rare native genetic resources.

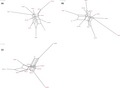

## 1 INTRODUCTION

Evidence has been found of horses present in Britain as early as the Middle Pleistocene, but horse and human populations seem to have become established following the last ice age during the middle Devensian period (Currant & Jacobi, 2001; Hosfield, 2011; Schreve, 2001). It is not clear when the ancestors of the modern native ponies of Britain and Ireland arrived, or how the currently recognized populations relate to each other in terms of their deep ancestral lineages. There are 11 recognized pony breeds of Britain and Ireland: the Welsh Pony and Cob, divided into four Sections A, B, C, and D according to height and conformational characteristics; the Fell and Dales breeds of Northern England; the Scottish Highland pony; the Eriskay pony of the Scottish Western Isles; the Shetland pony of the Shetland Isles; the Dartmoor, Exmoor, and New Forest breeds of Southern England; and the Irish breeds of Connemara and Kerry Bog Pony. Additionally, there is a recently characterized feral pony population living in the Carneddau mountain range of Snowdonia, North Wales (Winton et al., 2013).

There have been limited studies of the native British and Irish pony breeds that specifically investigated the genetic diversity within and between these populations in relation to each other. One study examined Irish breed maternal origins and mitochondrial diversity (McGahern, Bower, et al., 2006), and three further works investigated genetic diversity in British and Irish breeds present in North America (Prystupa, Hind, Cothran, & Plante, 2012; Prystupa, Juras, Cothran, Buchanan, & Plante, 2012; Winton et al., 2015). Bower et al. (2011) investigated the maternal origins of the Thoroughbred in context with a number of British and Irish pony breeds. Others have incorporated subsets of British and Irish pony breeds as part of a wider dataset for microsatellite or mitochondrial analysis, although the sample sizes were small and sample origins were often not stated or were from daughter export populations (Jansen et al., 2002; Luis, Juras, Oom, & Cothran, 2007; Leroy et al., 2009; Georgescu, Manea, Dudu, & Costache, 2011; van de Goor, Haeringen, & Lenstra, 2011). This study determines the genetic diversity and history of British and Irish pony breeds, by examining them as populations in their own right and in relation to each other.

Native pony breeds are part of British and Irish history and culture and represent an important source of uncharacterized genetic diversity. The adaptations to local conditions present within native ponies may provide an inimitable pool of genotypic characteristics of potential value to breeders of modern domestic horses. However, many of these breeds have suffered population decline and several are categorized as being “at risk” by the Rare Breeds Survival Trust (https://www.rbst.org.uk/rare-breeds-watchlist). Worldwide, pony populations that have historically undergone population reduction but have been subject to carefully managed conservation programs have shown success in improving their genetic viability. For example, following a 10‐year breeding management and conservation plan, the previously endangered Pottoka breed now displays high genetic diversity (Cañon et al., 2000; Rendo, Iriondo, Manzano, & Estomba, 2012; Solis et al., 2005). The Irish Kerry Bog Pony and the Scottish Eriskay pony have only been recognized as distinct breeds relatively recently (Beck, 1990; McGahern, Bower, et al., 2006). These are now considered to have ancient regional ancestry and as such are currently subject to breeding management and conservation efforts. Therefore, understanding the diversity within and between the British and Irish native pony breeds is essential to enact appropriate, informed conservation plans.

The aim of this study was to assess the genetic diversity, population structure, and gene flow of native ponies of Britain and Ireland using mitochondrial DNA and a panel of microsatellite markers. The dataset generated herein was also placed in context with other comparable datasets, including McGahern, Bower, et al. (2006), Achilli et al. (2012), Prystupa, Hind, et al. (2012) and Prystupa, Juras, et al., 2012).

## 2 MATERIALS AND METHODS

### 2.1 Sample collection and genomic DNA extraction

**Figure 1:**
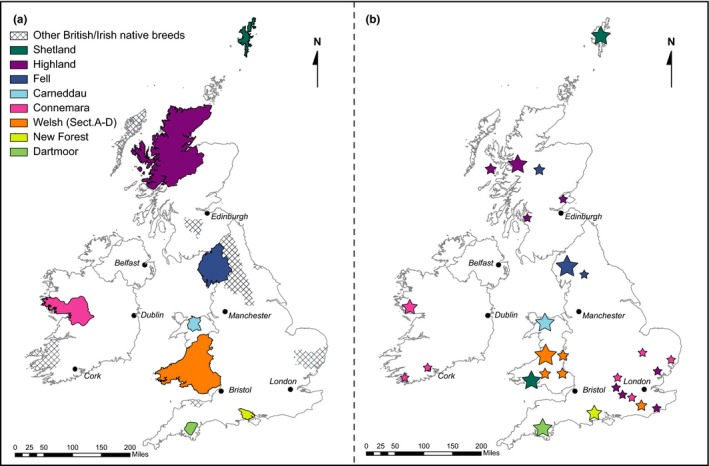
Approximate geographical origin of the British and Irish native pony breeds (a) and the location of samples collected for this study (b)

Studies were undertaken using animals representative of different bloodlines for each of eleven British and Irish native pony breed populations (Figure 1). DNA samples were collected as previously described for UK and Irish sources (Winton et al., 2013). A total of 485 animals were used for the mtDNA analysis and 450 for the microsatellite analysis (see Summary Statistic tables in relevant sections for population sample numbers).

### 2.2 Microsatellite genotyping

SSR genotyping of samples was performed for 17 SSR loci using commercially available kits and protocols as described previously (Winton et al., 2013). PCR products were sized using an ABI 3730 Genetic Analyzer (Applied Biosystems) with the internal 600‐Liz size standard. Genotypes were determined using Genemapper^®^ software v3.0 (Applied Biosystems).

### 2.3 Microsatellite analysis

Allele frequencies were examined for significant deviation (*p* < .05) from Hardy–Weinberg expectations using the exact test in Arlequin 3.5 (Excoffier, Laval, & Schneider, 2005; Excoffier & Lischer, 2010), with the Markov chain length set to 100,000 following 10,000 dememorization steps. Markers displaying highly significant deviations in more than one population and/or containing large amounts of missing data values were removed to limit the influence of allele dropout. Loci with missing data unevenly distributed across the populations (>5% per breed) were also excluded resulting in seven loci being removed from the analysis, including two that had demonstrated null alleles in another study (Rendo et al., 2012). Individual samples with poor quality DNA with calls missing from >3 loci were also excluded.

An unbiased Bayesian approach using Markov chain Monte Carlo (MCMC) clustering of samples was conducted via the STRUCTURE v2.2.3 software (Pritchard, Stephens, & Donnelly, 2000). Parameters were set as for diploid data for each individual and assessed for values of *K* ranging from 1 to 14. Burn‐in and MCMC iteration settings were 50,000 and 100,000, respectively. Allele frequencies were treated as correlated. For each value of *K*, six replicate simulations were conducted. The Δ*K* statistic (the second order rate of change in log probability [Ln Pr(X/*K*)] between successive values of *K*) was calculated using STRUCTURE Harvester v0.6.7 (http://taylor0.biology.ucla.edu/struct_harvest/) as per Evanno, Regnaut, and Goudet (2005). Results from replicate runs at the optimal *K* were combined in CLUMPP (Jakobsson & Rosenberg, 2007) and the average Q‐table exported to DISTRUCT (Rosenberg, 2004) for graphical presentation.

A matrix of classic genetic distance between individuals was calculated according to Nei's standard distance (*D*_s_) (1972) using Populations 1.2.31 (Olivier Langella; http://bioinformatics.org/~tryphon/populations/). This matrix was visualized by drawing an unrooted neighbor‐joining phylogenetic tree with MEGA 5.1 (Tamura et al., 2011). An additional distance matrix was calculated based on Reynold's unweighted genetic distance (Reynolds, Weir, & Cockerham, 1983) using Populations 1.2.31 (Olivier Langella; http://bioinformatics.org/~tryphon/populations/) and visualized by drawing a NeighborNet in SplitsTree 4.13.1 (Huson & Bryant, 2006).

Population summary statistics, pairwise *F*_ST_ comparisons, and analysis of molecular variance (AMOVA) were calculated using Arlequin 3.5 (Excoffier & Lischer, 2010). Pairwise *F*_ST_ comparisons were performed using two methods to calculate distance: one based on the number of different alleles and the other based on the sum of squared size difference, similar to the *R*_ST_ measure of (Slatkin, 1995). The *R*_ST_ calculation is a genetic distance method designed for SSR data, a measure of pairs of populations based on the stepwise mutation model (SMM) calculating the average number of allelic size differences between populations.

In order to account for difference in sample size, allelic richness (AR) was calculated for each locus and population based on a minimum sample size of 21 individuals using FSTAT v2.9.3.2 (Goudet, 1995).

### 2.4 mtDNA genotyping

UK samples were sequenced for a 606bp mtDNA fragment of the mitochondrial control region (MTCR) as described previously (Winton et al., 2013). Sequence data were trimmed to a common 519bp sequence and aligned using BioEdit Sequence Alignment Editor v7.0.9.0 (Hall, 1999) before being exported in PHYLIP (^*^.phy) format.

### 2.5 mtDNA analysis

A median‐joining network was created to explore the relationship between haplotypes using NETWORK (http://www.fluxus-engineering.com). In line with other studies, hypervariable nucleotide positions 15,585, 15,597, and 15,650 were removed (downweighted to 0) and nucleotide positions 15,604, 15,659, and 15,737 were downweighted (from 10 to 5). Nucleotide positions refer to that of the complete horse reference sequenced by Xu and Arnason (1994). The 83 reference sequences from Achilli et al. (2012) were downloaded to create a skeleton network of the globally defined horse haplogroups. Sequence traces were checked for nucleotide call errors and either manually rectified or removed in the case of overall low‐quality sequence.

Haplotype number, diversity, and nucleotide diversity were calculated for each population using DnaSP v5 (Librado & Rozas, 2009). A haplotype list was generated for each dataset by DnaSP v5 based on the original sequences and used in Arlequin 3.5 (Excoffier & Lischer, 2010) to calculate the average pairwise nucleotide differences between each population and to estimate number of migrants exchanged between populations. Mismatch distributions were also analyzed to detect evidence of population expansion.

## 3 RESULTS

### 3.1 Microsatellite analysis of native British and Irish pony breeds

#### 3.1.1 Population genetic diversity parameters

Summary statistics for microsatellite (SSR) data analysis are presented in Table 1. A total of 450 individuals were successfully genotyped. The mean number of alleles per locus (MNA) ranged from 5.92 to 7.58 averaged across all populations for the 12 SSRs, with mean expected (*H*_E_) and observed (*H*_O_) heterozygosity values of 0.721 and 0.659, respectively. The New Forest pony displayed the highest overall diversity, with the highest AR value (6.83) and *H*_O_ (0.731) and low *F*_IS_ (−0.016, heterozygote excess). The Section C had the lowest AR (5.61) and the highest *F*_IS_ (0.049, closely followed by the Carneddau at 0.048). The Shetland showed the lowest *H*_O_ at 0.680. The Section A also had low *H*_O_ (0.687) and relatively high *F*_IS_ (0.033), but had the second highest AR and MNA (6.42 and 7.25, respectively).

**Table 1:** Summary statistics for SSR genotyping for each of the 11 populations

	*N*	MNA	AR	*H*_O_	H_E_	*F*_IS_
Section A	53	7.25	6.42	0.687	0.715	0.033
Section B	25	6.42	6.17	0.685	0.726	0.020
Section C	34	5.92	5.61	0.705	0.736	0.049
Section D	46	6.58	5.81	0.697	0.713	0.017
Fell	39	6.17	5.73	0.692	0.716	0.001
Highland	44	6.67	6.01	0.728	0.718	−0.023
Connemara	48	6.58	5.99	0.718	0.746	0.033
New forest	41	7.58	6.83	0.731	0.728	−0.016
Dartmoor	38	7.00	6.40	0.698	0.702	0.005
Carneddau	47	7.00	6.14	0.705	0.740	0.048
Shetland	35	6.42	5.82	0.680	0.688	0.009
Total/mean	450	6.69	7.13	0.659	0.721	0.017*

Abbreviations: AR, allelic richness based on a minimum sample size of 21 individuals; *F*_IS_, inbreeding coefficient; *H*_E_, expected heterozygosity; *H*_O_, observed heterozygosity; MNA, mean number of alleles; *N*, sample number.

* Significant deviation from HWE at *p < *.05.

#### 3.1.2 Population structure and gene flow

Molecular analysis of variation demonstrated highly significant genetic structure within the dataset (*p* < .001), with 6.94% of the total variation existing between populations. Variation within individuals was 91.51%.

**Figure 2:**
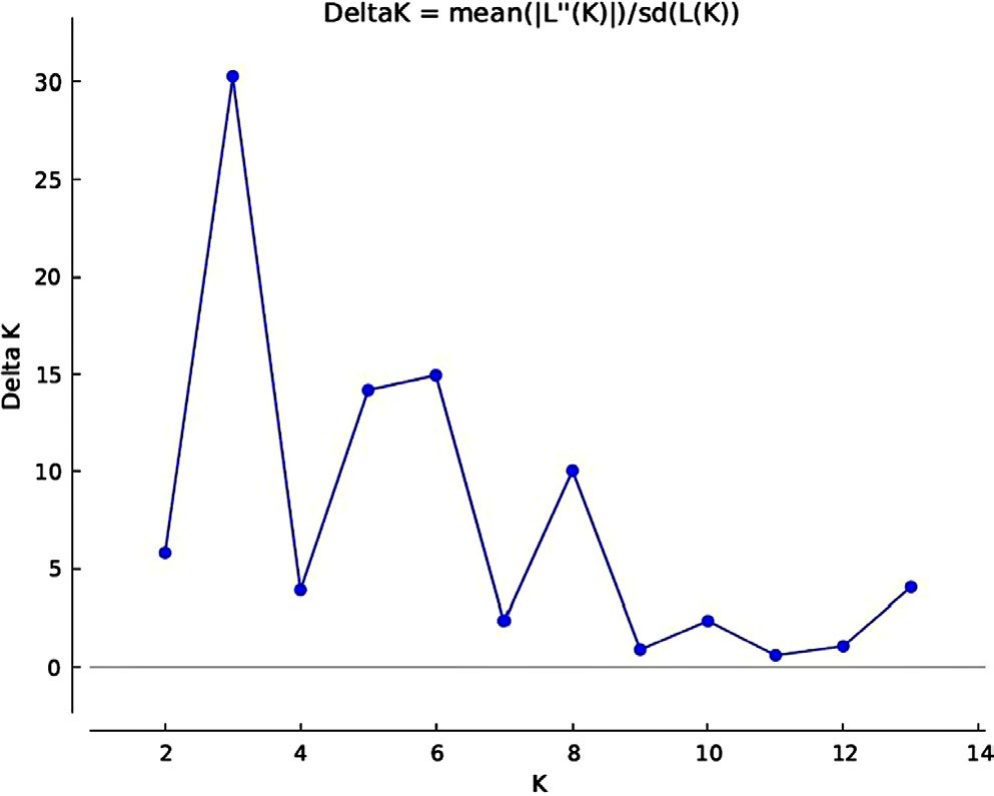
The ΔK plot, describing the rate of change in the log probability of the data between successive *K* values from 1 to 14. The modal value of this distribution is the true *K*, or the uppermost level of hierarchical structure, as per Evanno et al. (2005)

**Figure 3:**
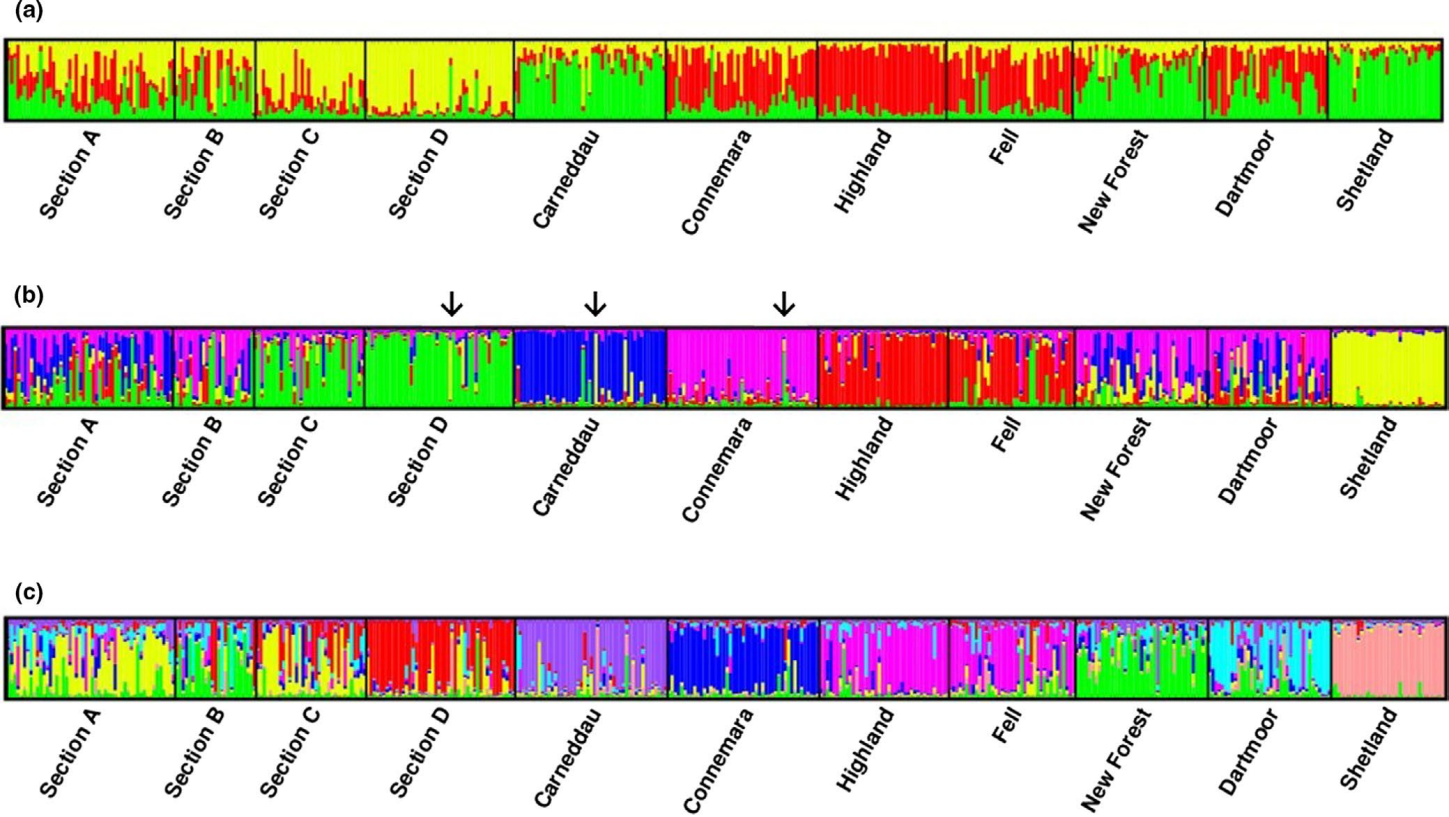
Consensus plots for six independent STRUCTURE analysis runs for 12 SSR loci of 11 populations for (a) *K* = 3, (b) *K* = 5, and (c) *K* = 8. Individual vertical bands depict single animals within a population, indicating the degree of genotype admixture between breeds according to shared allele frequencies. ↓ = examples of animals within a breed that were assigned to another cluster according to their allele frequency profile

STRUCTURE Harvester analysis suggested the uppermost hierarchical level of structure was three clusters, with probable subclusters at *K* = 5 and *K* = 8 (Figures 2 and 3; Evanno et al., 2005). In general, this analysis indicated no single clear structure in the data reflecting a complex history of population interactions rather than a simple history of population bifurcation. All populations contained individual animals that could not be unambiguously assigned to a single cluster (Figure 3). There were also misassigned individuals in populations; both Carneddau and Section D populations had animals with near 100% probability of assignment to the Shetland cluster; and a Connemara were similarly assigned to the Section D cluster. At *K* = 5 the feral Carneddau population, the Connemara and the Shetland showed distinct breed clusters, while the Sections C and D, and the Fell and Highland ponies clustered into two groups, respectively. When *K* was increased to 8, the Fell and Highland breeds remained in a single cluster and a high number of Section Bs showed high probability of assignment to the New Forest cluster. While for most other populations, specific genotypic signatures were evident, there was a relatively high degree of admixture between many of the pony breeds, particularly the Section A.

**Figure 4:**
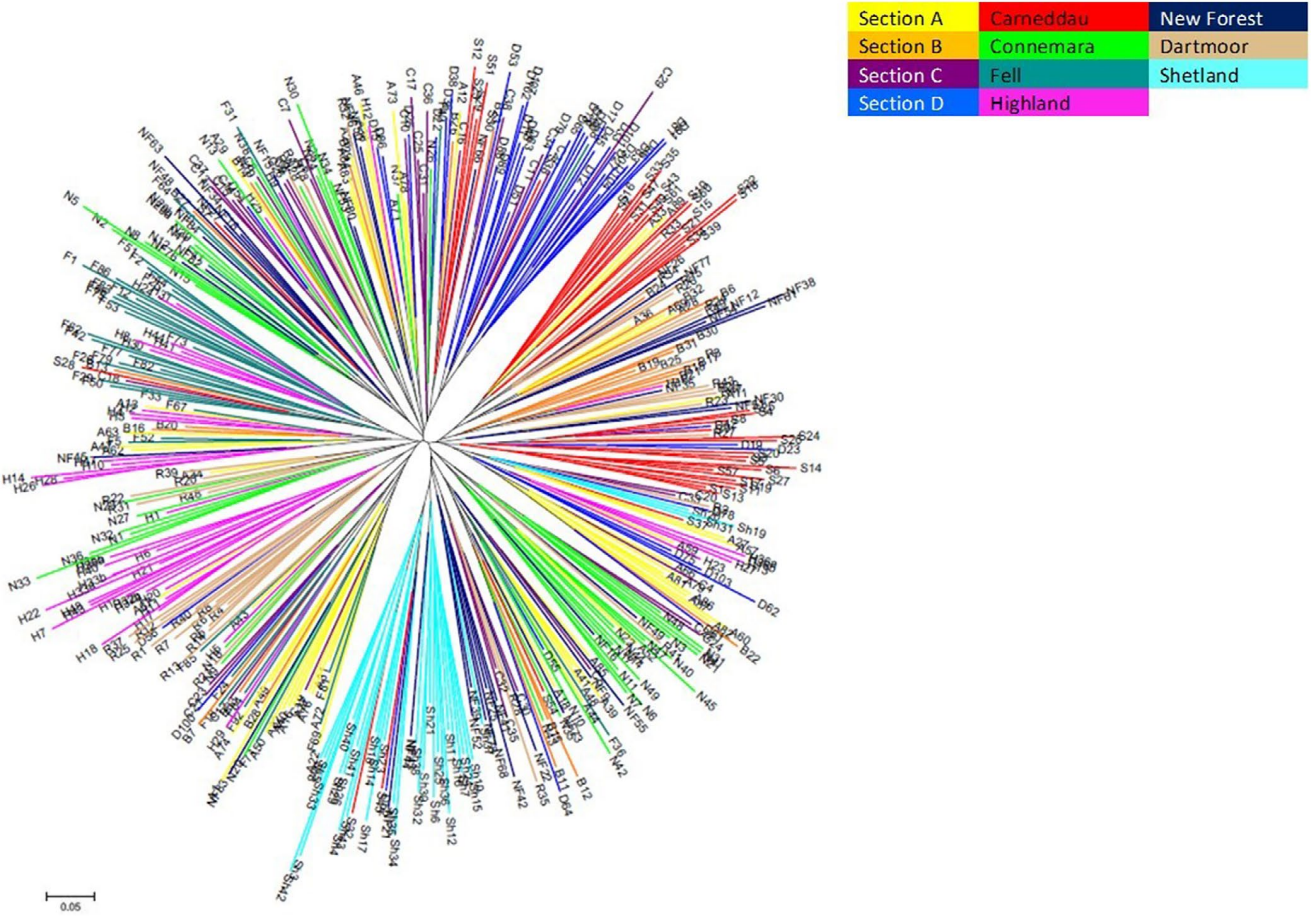
The unrooted individual neighbor‐joining phylogenetic tree based on Nei's standard distance (Nei, 1972). Branches are colored according to population

The unrooted neighbor‐joining phylogenetic tree based on Nei's standard distance measure (*D*_s_) (Nei, 1972) between all individual ponies demonstrated a variable degree of clustering according to breed (Figure 4). As also seen in the STRUCTURE plots, Shetlands showed the greatest breed‐specific clustering (Figures 3 and 4). The main Fell pony cluster contained a number of Highland pony samples, echoing the admixture between these breeds evident in the STRUCTURE plots. The Carneddau ponies split into two main clusters, also containing several Section As or Section Ds in each. Overall, the tree showed relatively high admixture between the pony breeds, a lack of deep structure in the phylogeny and most variation occurring between individuals.

**Figure 5:**
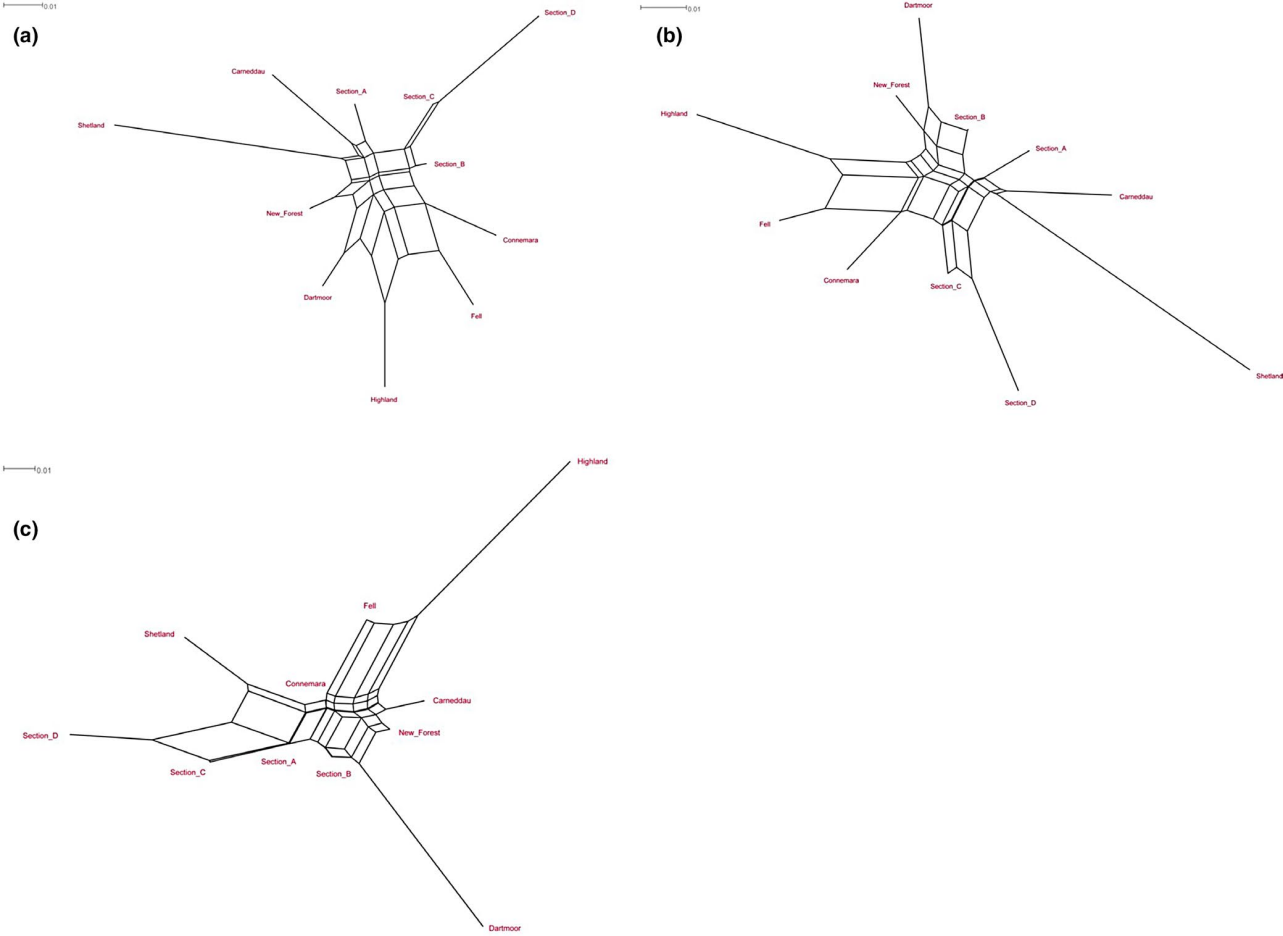
(a) NeighborNet drawn using a Reynold's unweighted genetic distance (Reynolds et al., 1983) matrix, based on the coancestry coefficient for populations diverging by drift only. (b) NeighborNet drawn using the pairwise *F*_ST_ comparison distance method, based on number of different alleles. (c) NeighborNet drawn using the pairwise *F*_ST_ comparison distance method, based on sum of squared size difference (*R*_ST_‐like)

All NeighborNet networks produced by SplitsTree using the different genetic distance matrices showed similar topologies, with a high degree of unresolved reticulation at the center of the network. This display of many alternative phylogenies indicates the relatively recent between‐breed admixture between British and Irish pony breeds. Many of the splits confirmed the clustering found according to Bayesian analysis (Figure 3). The Shetlands were the most diverged from the other breeds (Figure 5), while the New Forest and the Section B showed lower divergence from many populations. The Carneddau population also diverged considerably from any other breed. The Section C and Section D clearly showed a close relationship for all networks, as did the Fell and Highland ponies. Correspondingly, the estimated effective number of migrants (*N*_m_) was highest between the Section C and Section D, while the Highland shared the highest *N*_m_ with the Fell (Table 2). Both the New Forest and the Dartmoor had the highest effective number of migrants exchanged with the Section B. Theta H values were lowest for the Dartmoor and Shetland and highest for the Connemara.

**Table 2:** Effective number of migrants and ϴ_H_ (on the diagonal)

	Section A	Section B	Section C	Section D	Fell	Highland	Connemara	New Forest	Dartmoor	Carneddau	Shetland
Section A	**1.95**	7.68	7.64	3.69	4.22	2.96	4.49	5.21	4.34	5.96	2.41
Section B	19.23	**2.01**	7.81	4.29	4.10	2.79	6.12	10.38	7.96	5.51	2.42
Section C	11.9	12.99	**2.07**	10.95	6.04	3.24	5.51	4.02	3.56	4.25	2.39
Section D	4.13	2.89	13.73	**1.95**	3.42	1.72	2.85	3.00	2.13	3.20	1.88
Fell	5.06	3.77	3.43	2.40	**1.96**	4.40	4.86	3.91	2.69	2.91	1.62
Highland	1.84	1.44	1.00	0.79	4.37	**1.97**	3.18	2.93	3.48	2.26	1.65
Connemara	10.86	17.8	7.89	2.72	17.43	2.24	**2.14**	5.55	3.98	2.92	2.02
New Forest	6.73	27.56	3.38	1.52	4.73	2.28	9.56	**2.02**	6.51	4.74	2.56
Dartmoor	2.25	4.59	2.41	1.21	1.67	1.07	3.50	3.49	**1.89**	3.24	1.98
Carneddau	8.52	9.83	3.01	1.64	3.2	1.92	3.78	10.72	1.86	**2.10**	2.44
Shetland	6.14	3.44	2.71	2.52	3.12	1.46	3.00	3.21	1.02	3.70	**1.83**

Note

Effective number of migrants (*N*_m_) per generation, based on Slatkin, 1995. Above diagonal: Distance method calculated according to number of different alleles. Below diagonal: Distance method calculated according to sum of squared size difference (*R*_ST_‐like). **Diagonal**: Theta H estimates 4Neµ = (1/(1 − He)^2^) − 1.

### 3.2 Mitochondrial DNA analysis of native British and Irish pony breeds

#### 3.2.1 Population genetic diversity parameters

Summary statistics are presented in Table 3. Analysis of mitochondrial control region sequence from 485 individuals identified 91 haplotypes based on 83 variable nucleotide sites. Overall, haplotype diversity was high (0.965 ± 0.003) with relatively low nucleotide diversity (0.0189 ± 0.0003). The Carneddau possessed the lowest haplotype diversity of all of the populations and the lowest number (8) of haplotypes. The Shetland and Dartmoor breeds also showed low haplotypic diversity, with only 10 and 12 haplotypes found, respectively, and the Dartmoor having the lowest nucleotide diversity value for all populations. In contrast, the Section D and Fell groups had the highest number of haplotypes (25 and 20, respectively) and also the highest haplotypic diversity values, while the Fell had the highest overall nucleotide diversity.

**Table 3:** Summary statistics for MTCR genotyping for each population

Population	*N*	*H*	*h* ± *SD*	*π* ± *SD*
Section A	47	17	0.936 ± 0.016	0.0175 ± 0.0003
Section B	29	13	0.914 ± 0.028	0.0128 ± 0.0025
Section C	35	12	0.909 ± 0.022	0.0182 ± 0.0011
Section D	51	25	0.947 ± 0.014	0.0187 ± 0.0008
Fell	46	20	0.955 ± 0.010	0.0215 ± 0.0014
Highland	46	16	0.928 ± 0.016	0.0138 ± 0.0018
Connemara	46	19	0.903 ± 0.027	0.0142 ± 0.0015
New Forest	46	17	0.926 ± 0.017	0.0196 ± 0.0008
Dartmoor	41	12	0.859 ± 0.030	0.0099 ± 0.0018
Carneddau	51	8	0.787 ± 0.036	0.0142 ± 0.0016
Shetland	39	10	0.808 ± 0.047	0.0191 ± 0.0014
Total	485	91	0.965 ± 0.003	0.0189 ± 0.0003

Abbreviations: *h*, MTCR haplotype diversity; *H*, number of MTCR haplotypes; MTCR, mitochondrial control region; *N*, sample number; *SD*, standard deviation; *π*, MTCR nucleotide diversity.

#### 3.2.2 Population structure and gene flow

Significant genetic structure was apparent (*p* < .001), with 14% of the total variation found between populations. Table 4 demonstrates that the maternal lineages in the Section A, Section C, and Section D animals could not be distinguished, with the lowest average number of pairwise nucleotide differences for all populations examined. In contrast, the Welsh Section B population showed significant divergence (*p* < .001) from the other Welsh populations. The Section B group was not found to be significantly differentiated from the Irish Connemara pony, with a very low value for the average pairwise differences and correspondingly high effective number of migrants (*N*_m_) between the two populations. The Section B and the Dartmoor ponies also appear to share maternal lineages and were not significantly divergent from each other. The Carneddau were significantly differentiated from every other population analyzed (*p* < .001). The Shetland pony was also highly divergent, though demonstrated closer maternal links with the Fell breed than with any other population. The Fell pony breed shared maternal lineages with the Welsh Sections A, C, and D.

**Table 4:** MtDNA population average pairwise nucleotide differences, within‐population pairwise differences, and estimated effective number of migrants

	Section A	Section B	Section C	Section D	Fell	Highland	Connemara	New Forest	Dartmoor	Carneddau	Shetland
Section A	**9.12**	6.65	124.27	inf	19.29	8.43	12.33	7.57	2.57	2.54	3.58
Section B	0.63***	**6.66**	5.02	7.29	2.53	8.27	90.64	2.24	11.93	1.03	1.38
Section C	0.04	0.82***	**9.46**	inf	12.36	4.24	7.79	4.66	1.95	2.83	3.17
Section D	−0.10	0.62***	−0.02	**9.86**	17.54	7.96	14.69	7.78	2.67	2.61	3.99
Fell	0.25	1.81***	0.41***	0.29*	**10.46**	3.18	3.86	7.75	1.36	3.12	8.36
Highland	0.49**	0.43*	0.96***	0.54***	1.40***	**7.19**	13.2	4.74	4.47	1.05	1.74
Connemara	0.34***	0.04	0.52**	0.30*	1.16***	0.2**	**7.38**	3.12	4.56	1.18	2.19
New Forest	0.64***	2.00***	1.06***	0.64***	0.67***	0.92**	1.40***	**10.16**	1.48	1.48	3.98
Dartmoor	1.39***	0.23	1.78***	1.43***	2.89***	0.69***	0.69**	2.58***	**5.13**	0.64	0.88
Carneddau	1.65***	3.56***	1.48***	1.69***	1.45***	3.56***	3.20***	3.00***	5.07***	**7.73**	1.24
Shetland	1.32***	3.10***	1.53***	1.24***	0.62*	2.41***	1.95***	1.26***	4.17***	3.49***	**9.92**

Note

Above diagonal: The effective number of migrants (horses) per generation (*N*_m_), according to Slatkin and Hudson (1991), inf = infinity. The estimated value of *N*_m_ is calculated based on the assumption of populations at equilibrium between migration and drift, whereby *F*_ST_ = 1/(2M + 1), and where the two populations to be compared exchange migrants only with each other. *M* can thus be solved to give the absolute number of migrants (*N*_m_) exchanged between those populations in order to maintain the observed *F*_ST_ value between them. **Diagonal elements**: Average number of pairwise nucleotide differences within population (πX). Below diagonal: Corrected average number of pairwise nucleotide differences (πXY‐(πX + πY)/2 between populations, where π is the average number of nucleotide differences between two randomly chosen sequences. *p*‐values:

* <.05,

** <.01,

*** <.001.

**Figure 6:**
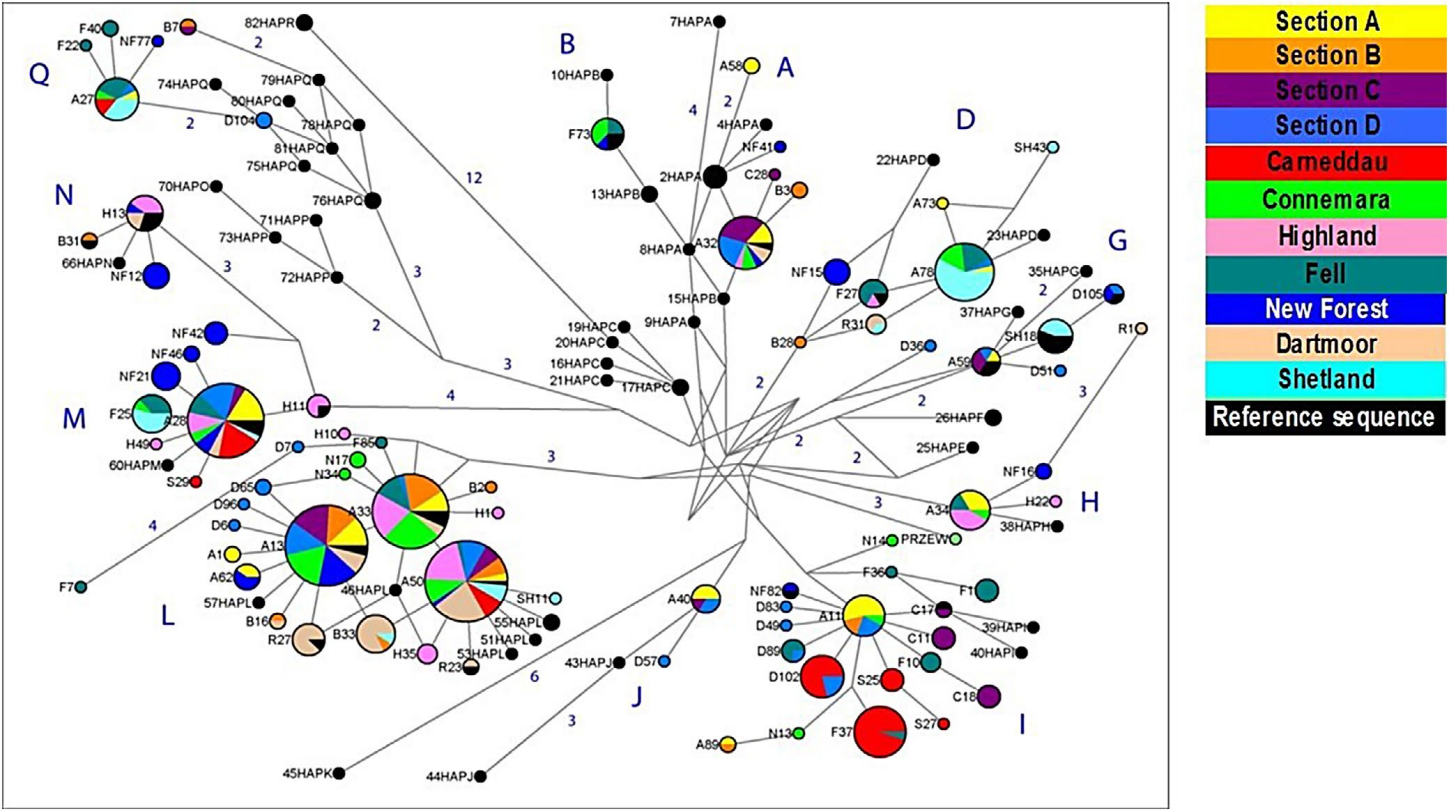
It shows a median‐joining network for mtDNA haplotypes based on shared mitochondrial haplotype frequencies. Node size represents overall haplotype frequency. The pie charts within nodes show frequency of that haplotype according to population (see population color legend). Reference samples representing examples of each of the major haplogroups identified by Achilli et al. (2012) are displayed in black and labeled according to sample number and haplogroup, for example, “1HapA.” Numerical values indicate the number of nucleotide changes (>1 mutation) between primary nodes/haplotypes. Branch lengths are proportional to the distance between nodes

The median‐joining network of Figure 6 displays the relationships of the maternal lines of the British and Irish pony breeds according to shared mitochondrial haplotype frequencies. Haplogroup I shows the star‐shaped pattern of an ancestral node with one‐ and two‐step mutations branching from it. This pattern was particularly evident for the Carneddau ponies, with unique or rare haplotypes present at high frequency within the population. The haplotype shared with the Section Ds (node "D102" within haplogroup I) is the result of a nucleotide insertion at 40nps (of the 519bp sequence analyzed) unique to these animals (three Section Ds and eleven Carneddau ponies). The other two Welsh populations Section B and Section C are also represented here, as well as a high presence of Fell ponies.

Some haplogroups consisted of a limited number of breeds, such as haplogroups J, G, N, and B. The node labeled “A40” is placed ancestrally to the J haplogroup reference sample (node "43HAPJ"), but after the J‐K haplogroup split. It is thus designated here as belonging to haplogroup J. This cluster was comprised solely of Welsh ponies and is not represented in any other British or Irish pony breeds. The G haplogroup contains a large number of Shetland individuals, as well as Welsh ponies (Sections A, C, and D) and a single New Forest, but no other breeds. Haplogroup N consists of predominantly Highland and New Forest. Haplogroup B is limited to (predominantly) Connemara, as well as two Fells and a New Forest.

**Figure 7:**
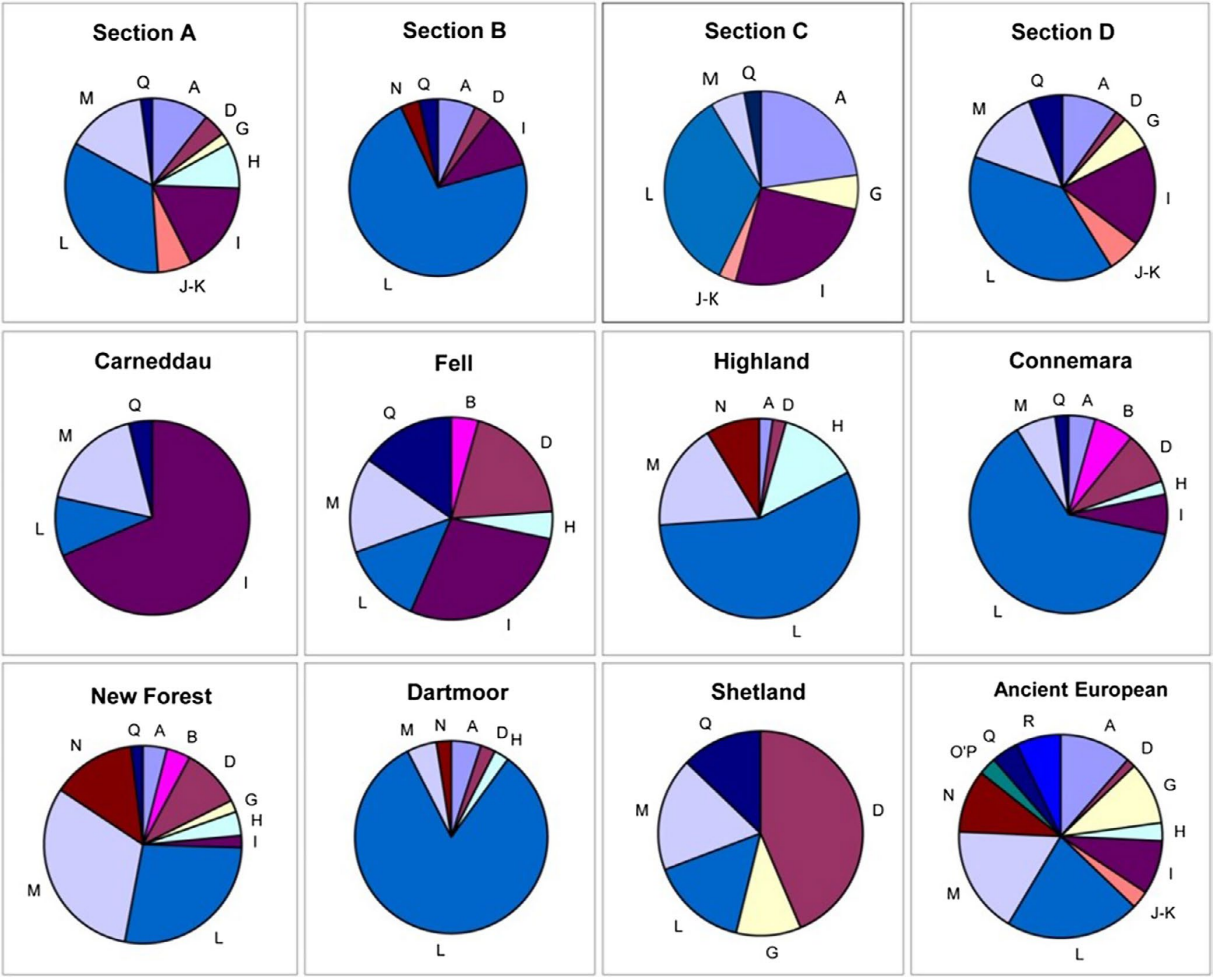
Haplogroup frequency (%) according to breed. Segments represent proportion of individuals belonging to labeled haplogroup

Certain breeds are particularly noteworthy due to their diversity patterns across the haplogroups (Figure 7 for haplogroup frequencies according to breed). The Carneddau contain the lowest number of haplogroups (and haplotypes), and 69% of samples have haplogroup I. The Section C and the Shetland similarly have more limited haplogroup diversity (six and five haplogroups, respectively), and the Shetland has a high frequency of individuals that are within haplogroup D (44%). Most breeds are represented within the highly shared nodes of haplogroup L (>27% of each breed are L haplogroup; comparable with the 38% L haplogroup frequency shown in European horses in Table 5). However, the Shetland, Carneddau, and Fell ponies have much lower L haplogroup frequencies than the rest of the breeds sampled here and other European horses (Table 5). In contrast, the Dartmoor breed is vastly overrepresented for haplogroup L, with 83% of Dartmoor ponies possessing this haplogroup. The Section B is also overrepresented for haplogroup L, with 72% of animals containing haplotypes from this group.

**Table 5:** Haplogroup frequency (%) according to global region of horse breeds

	A	B	C	D	E	F	G	H	I	J‐K	L	M	N	O'P	Q	R	Sample numbers
European	4.49	9.38	0.32	4.57	0.48	—	8.73	1.04	8.01	0.56	38.06	7.29	8.49	1.36	3.85	2.24	1,249
Middle Eastern	7.81	10.94	3.13	2.08	0.52	—	9.09	3.03	15.15	—	24.24	3.03	3.03	9.09	9.09	—	192
Asian	11.93	1.70	3.58	2.90	2.21	3.07	16.35	1.36	6.13	6.47	13.46	4.09	2.90	6.64	13.80	1.87	587
Ancient European	11.43	—	—	1.43	—	—	10.00	2.86	8.57	2.86	21.43	17.14	10.00	2.86	4.29	7.14	70
Ancient Asian	8.82	—	—	10.29	—	1.47	22.06	5.88	2.94	2.94	10.29	2.94	4.41	10.29	10.29	7.35	68

Note

Haplogroup frequency (%) according to geographical region, as described by Achilli et al. (2012). The first row identifiers “A” to “R” represent the major worldwide mtDNA haplogroups in horses. Haplogroup classification is based on control region motifs. Geographic regions are designated according to sequence data collated by Achilli et al. (2012) and in total comprise of 2,166 reference sequences, including ancient (fossil) DNA data.

The New Forest is the most diverse group; it has animals represented within all haplogroups except the ancestral J node that is restricted to the Welsh populations. Many breeds frequently shared haplotypes with other breeds within each haplogroup. However, the New Forest had one or more breed‐specific haplotypes within most haplogroups, including clusters of multiple animals. However, there was a distinct lack of intermediate nodes connecting these unique clusters; that is, the New Forest ponies were poorly represented within, or missing from, the nearest “shared” haplotype node. In contrast, the Section A showed few breed‐specific haplotypes yet also had representative animals within most haplogroups (all except B and N). However, most examples of shared haplotypes for this breed occurred in the more ancestral nodes for each haplogroup.

## 4 DISCUSSION

The results revealed lower autosomal genetic differentiation according to breed (8%) compared to previously reported data of other horse breeds worldwide, in which 10%–17% of the genetic diversity could be partitioned this way (Aberle, Hamann, Drögemüller, & Distl, 2004; Glowatzki‐Mullis et al., 2006; Plante et al., 2007; Prystupa, Juras, et al., 2012). In part, this may be because this study consists solely of pony breeds originating from Britain and Ireland and recent interbreeding must be considered more widely during their historical development than the more distantly related breeds analyzed in other studies. The historical admixture demonstrated between British and Irish ponies is evident for all analyses conducted. For example, the high within‐population diversity and mixed individual probability of assignment for the STRUCTURE plots and the unresolved internal structure of the NeighborNet networks according to different distance analyses indicate the lower divergence and among population allele sharing.

Heterozygosity levels for British and Irish ponies, although lower than some existing reports (Leroy et al., 2009; Prystupa, Juras, et al., 2012), remain relatively high and are comparable to values in other European pony breeds, including semiferal ponies (Luis et al., 2007; Rendo et al., 2012; Solis et al., 2005). Mitochondrial diversity was very high in British and Irish ponies, with ponies representing almost all of the global horse haplotypes (Jansen et al., 2002). Mitochondrial sequencing shows extensive sharing of haplotypes across all the populations analyzed here and with those from other studies (Achilli et al., 2012; Prystupa, Hind, et al., 2012). This is a likely legacy of extensive crossbreeding occurring historically between populations of horses and ponies prior to recognized “closed” breed formation (McGahern, Edwards, et al., 2006; Vila et al., 2001). However, there is more breed‐specific structure in the mtDNA (14% compared with 8% autosomal differentiation) and breed‐specific haplogroups were found, such as haplogroup B in Connemaras and haplogroup N in Highlands. This likely reflects the human derived practice of bringing stallions from different locations or breeds to “improve” local populations of resident mares. The I haplogroup is interesting in that it shows a more established pattern of increased derived mutations than displayed by the Jansen et al. (2002) network and exists in high frequency in several British and Irish native breeds. This haplogroup is predominantly found in Welsh ponies and Fell ponies. The star‐shaped pattern of expansion and the unique haplotypes seem to indicate an ancestral presence (Kavar & Dovc, 2008) long established within the ponies of the British Isles, and the Welsh ponies in particular. The root node containing pony samples within this dataset was placed ancestrally to the reference samples within this network. This perhaps indicates older origins for the ancestral British and Irish native ponies than those of the apparently derived haplotypes of the reference sequences used by Achilli et al. (2012) to define the haplogroups.

The exclusive presence of Welsh ponies in the "A40" node, placed ancestrally to the J haplogroup reference sample, is also particularly interesting. This haplogroup is rare in modern European horses, although is prevalent in higher frequency in ancient European and Asian samples. The node for the J‐K haplogroup split is estimated to date back to between 60 and 80kya, and the J haplogroup is estimated at approximately 20kya. The A40 haplotype shared by the Welsh ponies (and one derived haplotype, D57) is placed between these nodes within the network. Therefore, Welsh ponies appear to be established from an ancient ancestral population, with some rare matrilines maintained within the population to this day. The presence of this node suggests the divergence of the ancestral stock during the periglacial period and that today's British ponies may represent a relic of the expansion of animals following the retreat of the last glacial period rather than more recent introductions in the historical period. Jansen et al. (2002) suggested a postglacial common origin for the Icelandic, Norwegian and Shetland pony breeds following the identification of an exclusive cluster, denoted E. This cluster corresponds to haplogroup D in this study, and although it is certainly predominated by the Shetland (and again contains a large number of Fells), there are also a few representatives from nearly every other population.

The Shetland pony showed evidence of more restricted diversity for mitochondrial analyses than other breeds. It is highly diverged from other pony breeds of Britain and Ireland for both autosomal and mitochondrial analyses and has been found to cluster with Nordic breeds, indicating a predominantly Scandinavian origin or influence (Bjornstad & Roed, 2001; Petersen et al., 2013; Prystupa, Hind, et al., 2012; Prystupa, Juras, et al., 2012). However, it is notably more closely related maternally to the Fell than to any other British or Irish breed analyzed here, with reduced pairwise differences and a high number of shared haplotypes. It is possible that the isolated Fell ponies of northern England shared and maintained ancestral links due to an ancient migration of ponies through Britain northwards to the Shetland Isles (Russell, 1996). Alternatively, a shared ancestral population of ponies transported by the Vikings may have been introduced to both regions during this period of extensive human settlement (Bowden et al., 2007; Goodacre et al., 2005; Richardson, 1990). This relationship has since diverged for the more rapidly evolving SSR markers. The drift shown here corresponds to the geographical location of the origin of these ponies; the Shetland Isles are a collection of remote islands located some 100 miles north of Scotland and 200 miles west of Norway. Many pony breeds suffered a population decline during the World Wars and economic depression, including the Shetland pony. However, the demand for these ponies may have remained comparatively more stable than other breeds due to the continuation of subsistence agriculture on the Shetland Islands, use within mines, and steady export to America and the Netherlands (Beck, 1990; Dent & Goodall, 1988; Russell, 1996). The restrictions in mitochondrial haplotype diversity appear to be the result of limited ancestral founding mares rather than any recent decline, and nuclear diversity is comparable to other British and Irish breeds. The census number of Shetlands is high within the UK as they are a well‐known and popular breed and are not considered to be at risk. If a wide range of mares and stallions continue to be used for breeding to ensure conservation of ancestral lines and continuity of gene flow, there is no risk to the genetic integrity of the Shetland pony breed.

The Dartmoor ponies demonstrated low mtDNA diversity but moderate to high nuclear diversity. This pattern is unusual in horse breeding within a closed population (Prystupa, Hind, et al., 2012; Prystupa, Juras, et al., 2012), as normally sex‐biased skew results in limited sire numbers and higher number of effective females. It appears that a bottleneck occurred in the ancestral Dartmoor population, followed by introductions from outside stallions to increase the population size. The relatively high degree of admixture seen for a number of Dartmoors according to SSR analysis supports this. Until the end of the 19th century, a wide variety of outside influences were introduced to Dartmoor stock, including Welsh Ponies and Cobs, Hackneys, Arabs and Barbs, small Thoroughbreds, and other native ponies (Edwards, 1992). These introductions largely coincided with the increased popularity of the sport of Polo, which resulted in crossing of Thoroughbred and Arab sires with native pony mares (Palmer, 1990). Other studies have also found that outcrossing usually occurs through the male (Pirault, Sophy Danvy, Verrier, & Leroy, 2013). The mating of mares from limited lineages may have occurred during the following population expansion, resulting in the overrepresentation of haplogroup L seen in the modern Dartmoor pony. It is also possible that Dartmoor mares descend from the ancestral population of modern Iberian stock, given the similarity in haplogroup frequency patterns and that Exeter has been an established port town since Roman times (Edwards, 1992). Theta H values, as analyzed for the SSR dataset, were reduced for the Dartmoor, indicating a lower number of effective breeders than the other pony populations. The Dartmoor ponies would benefit from careful breeding management in order to preserve the limited mitochondrial diversity and rare matrilines. These individuals might be identified through comparison of mitochondrial results with genealogical records to allow for breeding selection accordingly.

The striking lack of distinction between the Section A, Section C, and Section D populations in matrilineal diversity suggests that they originate from the same ancestral population of mares. Extensive interchange of mares between the populations is excluded by the clear contrast in the nuclear data for the Section A and Section D. The distinct differentiation in the SSR plots in combination with similarity in mtDNA implies extensive male‐biased line‐breeding. There is historical evidence of multiple importations of foreign stallions to breed with local Welsh mares and that this influenced the development of the Section D (Davies, 1993, 1999; Welsh Pony & Cob Society, 2002). The central position of the Section A animals in the NeighborNet plots would be consistent with the assumptions that this breed has retained many ancestral allele frequencies. Alternatively, it may well reflect the influence that Welsh ponies have had on the development of many other breeds, resulting in maintained shared allele frequencies between these populations and the Section A. In contrast, the other native breeds have diverged in different directions to each other because of differential selection and specialization during breed formation. The high degree of admixture shown within the STRUCTURE plots and the N‐J tree according to the SSR data substantiate this. It is known that Arab and Thoroughbred stallions were introduced to Welsh hill pony mares in the 18th and 19th centuries and some of the individual variation within the Section As may reflect the presence of these alleles (Edwards, 1992). The census population for Welsh Ponies and Cobs is high both within the UK and globally, so as a breed they are not at risk. However, they do show lower observed heterozygosity and a higher inbreeding coefficient than other native breeds, which is likely to be a reflection of use of fewer sires in more recent times. Increasing the pool of sires for breeding (rather than limiting it according to popular champion animals) would be better for the genetic viability, particularly for the rarer Section C animals. The practice of breeding from high numbers of mares should continue to preserve the high ancestral diversity and rare matrilines found in the Welsh Ponies and Cobs. The genetic signature shown by the Section A pony may also be a result of Wahlund effect, whereby the formation and divergence of unrecognized subpopulations result in an overall reduction in observed heterozygosity compared to that predicted from allele frequencies calculated overall (Allendorf, Luikart, & Aitken, 2013). The Section A pony group shows high allelic richness and mean numbers of alleles, but has a heterozygote deficit (lower *H*_O_ and moderately high *F*_IS_). This phenomenon has been seen in other species such as goats, chickens, and wild sheep (Barker et al., 2001; Pham et al., 2013; Worley et al., 2004). STRUCTURE plots showed a variable probability of assignment of the Section A with a number of other populations, but no obvious within‐population subclusters. However, there were some small subclusters of Section A individuals present within mixed groups of individuals for the N‐J tree. It is possible that cryptic population substructure within the Section A has resulted in lower total heterozygosity for the population. This may result from selective breeding of lineages for different phenotype, for example, the hardy, stockier pit ponies versus more morphologically lightweight lines used for competitive showing, with greater Arab influence. Pirault et al. (2013) found evidence of high *F*_IS_ and Wahlund effect through examination of the pedigrees of Welsh Ponies and Cobs, though it is not clear whether the Welsh Pony group had been divided according to Sections A and B.

It is particularly interesting that the Welsh Section B is not mitochondrially distinct from the Dartmoor pony or the Connemara, yet is highly divergent from the other Welsh populations. There is some maintained relationship with the Dartmoor for the autosomal data, but no more than the relationship with the Section A and Section Cs. The Section B has clearly had a different maternal history relative to the other Welsh pony populations. The modern Section B Welsh Pony was developed into a taller, “more refined” riding pony from the Section A Mountain Pony and included considerable influence from Arab and Barb horses (Edwards, 1992). Both Arabs and Welsh ponies have had a significant influence on the development of the New Forest breed (Edwards, 1992); thus, the genotype sharing between the Section B and the New Forest displayed in the STRUCTURE plot at *K* = 8 is not surprising.

Despite considerable demographic changes over the last 135 years, the New Forest breed has high mitochondrial diversity. It is possible that this is due to the traditional husbandry methods of the free‐living mares within the Forest (while stallions have been more rigorously selected) https://www.rbst.org.uk/new-forest-pony, which results in lower reproductive variance than other managed breeds in a similar manner to the Carneddau ponies. The history of the New Forest breed and examination of pedigree lines of the unique nodes appears to support the theory of a relatively recent and severe bottleneck, followed by an expansion. This extinction of ancestral shared haplotypes within the New Forest, followed by the expansion of the remaining unique haplotypes, would explain the pattern seen whereby a single individual is present within a haplogroup otherwise exclusive of the breed (e.g., haplogroups Q and I). The admixture shown for the SSR STRUCTURE plots, the lack of breed‐specific clustering in the individual N‐J tree, and the low divergence also confirms the mixed heritage of the New Forest ponies for the autosomal data. Prystupa, Juras, et al. (2012) and Van de Goor et al. (2011) found similar genetic patterns for the New Forest breed. Petersen et al. (2013) similarly found high diversity and low inbreeding for the New Forest and also considered this was an effect of the more open management of this breed. However, there is a defined breed‐specific genotype cluster for the STRUCTURE plot at *K* = 8, albeit also shared with a considerable number of Section Bs. A variety of other native ponies were introduced to the New Forest during breed development, prior to the stud book closure in the 1930s, including recognized foundation stallions that contained considerable Welsh heritage (Edwards, 1992).

Fell ponies displayed very high maternal diversity, with a broad distribution of haplogroups, and moderately high nuclear diversity. Fell ponies, like many other native breeds, suffered population declines following the increased use of motor vehicles and mechanized farming in the early 1900s. Despite this, traditional shepherding of the fell uplands continued to some extent, as did limited keeping of free‐roaming herds on the mountains (Richardson, 1990). The Fell has shared maternal lineages with Sections A, C, and D, and to a lesser extent a relationship with the New Forest and Shetland. This is in contrast to the SSR data, where the Fells show the closest relationship to the Highland. According to Richardson (1990), up until the late 19th century the name “Galloway” was used indiscriminately for members of both an old Scottish breed (now extinct) and the Fell ponies prior to their official breed name (decided in 1898), and a shared connection between the two populations previously theorized (Beck, 1990; Dent & Goodall, 1988; Richardson, 1990). It is likely given the results found here, that sharing of stock occurred between these regions, with introductions of ponies of Fell‐Galloway origin into the Scottish Highlands via drovers, thus influencing the development of the modern Highland pony breed. It is apparent that this happened largely according to male‐biased crossing, as the maternal data do not show significant haplotype sharing between the Highlands and Fells.

The Fell ponies showed the closest autosomal relationship with the Welsh Ponies and Cobs (particularly Section C), the Connemara, and the Highland. The Fell and the Welsh Sections share a strong phenotypic trait of a notably fast trotting ability. For both populations, trotting races were a particularly popular activity in the 1800s and stallions were recorded to have been shared between the breeds (Lewis, 1988; Richardson, 1990). The Fell and the Welsh populations have maintained the greatest maternal diversity of the ancestral British ponies, with the presence of rare ancestral haplotypes and unique derived haplotypes. The retention of ancient mitochondrial diversity is likely a function of the isolated nature of these upland ponies and the fact that foreign introductions have largely been limited to male‐biased influences. The Fell pony numbers have increased over the last ten years, with the breed now placed within the “at‐risk” category of the Rare Breeds Watchlist (900–1,500 registered breeding females; https://www.rbst.org.uk/watchlist.pdf). The Fell breed suffers from foal immunodeficiency syndrome (FIS), a fatal autosomal recessive disease. The breed society currently recommends the practice of breeding FIS clear ponies with carriers and testing the offspring for FIS status in order to avoid genetic degradation (Carter, Fox‐Clipsham, Christley, & Swinburne, 2013; http://www.fellponysociety.org.uk/health.htm). It is important that this practice is adhered to (rather than avoiding breeding carriers entirely) in order to maintain the extensive maternal diversity within the Fell breed.

Despite the Connemara developing as a native pony on a neighboring island country, it shows no greater divergence than between British origin native breeds and indicates the extent of historic migration and exchange of animals across the Irish Sea. Welsh, Thoroughbred, and Arab animals are known to have influenced the development of the modern Connemara pony (Edwards, 1992). The genetic relationship with the Section Bs (also influenced by Arabs and possibly Thoroughbreds) is evident for both the mitochondrial and the autosomal data. The Connemara and the Highland pony bear the closest relationship for the mitochondrial data, yet the Highland is clustered more closely to the Fell pony according to nuclear analyses. This is in contrast to the results reported by Alderson (2005), whereby Highlands were not found to cluster with any other British pony breed, but were more closely associated with draught horses such as the Scottish Clydesdale. In this case however, the Highland and Fell do not separate into separate clusters until *K* = 9, indicating strong sharing of alleles between these breeds. Interestingly, while Petersen et al. (2013) did not examine Highland ponies, the Fells were found to cluster with several draught horse breeds, including the Clydesdale. This may thus be a reflection of historical gene flow between these three breeds, with the Highlands as an intermediary. The results of this study suggest conclusively that the maternal and paternal histories of the British and Irish pony breeds studied here are distinct and indicate a shared deep relationship beyond the historical period.

## 5 CONCLUSIONS

This is the first study that specifically investigates the genetic diversity within and between the British and Irish native pony breeds using large sample numbers from locations of their native origin. The state of the genetic diversity and the interrelationships of native British and Irish ponies were revealed following a combined mitochondrial and autosomal analysis approach. Overall, native ponies have maintained a high degree of genetic diversity, although small or isolated populations such as the Shetland, Carneddau, and Section C showed some reduction. While there were some unusual mitochondrial diversity distribution patterns, such as for the Carneddau and Dartmoor, there was a high degree of haplotype sharing among breeds and global haplogroups which were well‐represented within British and Irish ponies. Most populations have maintained much ancestral maternal diversity, but particularly the Fells and Welsh ponies, which display rare and ancient lineages. The maternal and paternal histories of the breeds are clearly quite distinct, with evidence of male‐biased crossings between native breeds and other shared influences, likely to include Arabs and Thoroughbreds.

Genetic studies that assess diversity and characterize origins of population are thus valuable tools in the development of conservation strategy. In this regard, the data herein indicate that for many of the native breeds, the breeding programmes should be maintained according to their current strategies as they are successfully sustaining their inherent genetic diversity. The Fell ponies face the challenge of reducing the proportion of FIS carriers in the population, but great care must be taken to ensure unique ancestral maternal haplotypes are not lost in the process. The Welsh A, C, and D also have a number of rare ancestral matrilines; however, it is important that as many quality sires as possible are included to maximize their nuclear genetic diversity. Additionally, this study recommends that Dartmoor breeding programmes should be carefully managed to preserve diversity and limited matrilines. This study has afforded valuable insight into the genetic diversity, the origins, and intricate interrelationships of most of the native pony breeds of Britain and Ireland.

## CONFLICT OF INTEREST

There are no conflicts of interest between authors, and permission from each author has been granted. No other institutions hold copyright over this work.

## AUTHOR CONTRIBUTIONS

Clare Winton conducted sample collection, laboratory and data analyses, and interpretation as well as writing of the manuscript. Robert McMahon provided invaluable input to data analysis approach and conducted some of those analyses, advising on their interpretation. Mathew Hegarty supervised sample analyses in the laboratory, data analyses, and interpretation. Neil McEwan oversaw the experimental setup (including research grant writing), cosupervised the student, and proof‐read and refined the manuscript. Mina Davies Morel provided historical context and interpretation of the data. Charly Morgan supported all laboratory analyses. Deborah Nash devised the study, wrote grant‐funding applications, supervised the student, and assisted with writing the manuscript.

## Data Availability

Mitochondrial sequence data generated as part of this project are stored in FASTA format and will be uploaded to the NCBI/GenBank nucleotide sequence repository. Other mtDNA sequences incorporated into the analysis were downloaded from this source and can be retrieved as per the relevant citations. Raw and processed SSR data trace files, and allele calls have been uploaded to DRYAD (https://doi.org/10.5061/dryad.63xsj3tzk). These data were converted into several formats for use in packages such as STRUCTURE; should access to each individual format be deemed necessary, they will also be stored on the DRYAD repository.
